# Impact of congenital heart disease on outcomes among pediatric patients hospitalized for COVID-19 infection

**DOI:** 10.1186/s12887-023-04058-2

**Published:** 2023-05-16

**Authors:** Laxmi V Ghimire, Fu-Sheng Chou, Othman A. Aljohani, Anita J. Moon-Grady

**Affiliations:** 1 Division of Pediatric Cardiology, Department of Pediatrics, University of California, San Francisco, Fresno, CA USA; 2 Department of Neonatal-Perinatal Medicine, Kaiser Permanente Riverside Medical Center, Riverside, CA USA; 3 Division of Pediatric Cardiology, Department of Pediatrics, University of California, San Francisco, 550 16th Street 5th Floor, San Francisco, CA 94158 USA

**Keywords:** COVID-19, Congenital heart disease, Children, Cardiovascular complications, United States

## Abstract

**Background:**

COVID-19 infection is generally regarded as an acute self-limiting illness in children, but it can cause significant morbidity and mortality in both healthy and high-risk children. There are limited data on the outcomes of children with congenital heart disease (CHD) and COVID-19. This study aimed to examine the risks of mortality, in-hospital cardiovascular and non-cardiovascular complications in this patient population.

**Methods:**

We analyzed data from hospitalized pediatric patients from 2020 using the nationally representative National Inpatient Sample (NIS). Children hospitalized for COVID-19 were included, and weighted data were used to compare in-hospital mortality and morbidities between children with and without CHD.

**Results:**

Out of 36,690 children admitted with a diagnosis of COVID-19 infection(ICD-10 code:U07.1 and B97.29) during calendar year 2020, 1240 (3.4%) had CHD. The risk of mortality in children with CHD was not significantly higher than those without CHD(1.2% vs. 0.8%, p = 0.50), with adjusted OR (aOR) of 1.7 (95% CI: 0.6–5.3). Tachyarrhythmias and heart block were more likely in CHD children with an aOR of 4.2 (95% CI: 1.8–9.9) and aOR of 5.0 (95% CI: 2.4–10.8), respectively. Similarly, respiratory failure [aOR = 2.0 (1.5–2.8)], respiratory failure requiring non-invasive mechanical ventilation [aOR = 2.7 (1.4–5.2)] and invasive mechanical ventilation [aOR = 2.6 (1.6-4.0)], and acute kidney injury [aOR = 3.4 (2.2–5.4)] were all significantly higher among patients with CHD. Median length of hospital stay in children with CHD was longer than those without CHD [5 days (IQR: 2–11) vs. 3 days (IQR: 2–5), p = < 0.001].

**Conclusions:**

Children with CHD hospitalized with COVID-19 infection were at increased risk of serious cardiovascular and non-cardiovascular adverse clinical outcomes. They also had increased length of hospital stay and utilization of healthcare resources.

**Supplementary Information:**

The online version contains supplementary material available at 10.1186/s12887-023-04058-2.

## Background

The coronavirus pandemic resulting from cases initially reported in Nov-Dec 2019 has affected people worldwide, with more than 600 million infections and more than 6 million deaths as a result as of November 2022 [1]. COVID-19 infection is generally regarded as an acute self-limiting illness in healthy children, but it can cause significant morbidity and mortality in both healthy and high-risk children [2]. Multi-system inflammatory syndrome in children (MIS-C) and other complications related to cardiovascular and non-cardiovascular systems have been reported in case reports and case series [3–15]. There have been reports of myocardial injury amongst children and adults hospitalized with severe COVID-19 infection. Possible causes of myocardial injury in patients with COVID-19 include myocarditis, hypoxic injury, cardiomyopathy, ischemic injury caused by cardiac microvascular damage or coronary artery disease. Given the risk of COVID-19 infection in children, vaccines against COVID-19 have been approved for use in children aged 6 months and older, and have been shown to be effective in preventing complications and hospitalization from COVID-19 infection [16–19].

Congenital heart disease (CHD) is prevalent in 1% of the population [20]. These children are at increased risk for complications from respiratory viral infections, including influenza and respiratory syncytial virus (RSV) [21, 22], but there are only limited data on complications associated with COVID-19 infection among children with CHD [2, 23, 24]. Here, we report an analysis from a recent nationwide cross-sectional retrospective study based on the United States National Inpatient Sample (NIS) database to examine mortality risks and in-hospital cardiovascular and non-cardiovascular complications as a result of COVID-19 infection among children with CHD.

## Methods

We analyzed hospital discharge records of nationally representative National Inpatient Sample (NIS) during the calendar year 2020 [25]. NIS is the largest publicly available all-payer inpatient healthcare database in the United States (US). The NIS database is compiled by the Agency for Healthcare Research and Quality (AHRQ) and is a part of the Healthcare Cost and Utilization Project (HCUP). NIS database is a stratified, cross-sectional database that accounts for ~ 20% of all discharges from acute care hospitals in the US and contains anonymized data on primary and secondary discharge diagnoses and procedures. The NIS dataset includes sampling weights that help to reduce the margin of error and sampling bias due to its multi-state nature and is estimated to represent more than 98% of hospitalizations in the US. Detailed description of the database is available at HCUP website [25]. This study included individuals (≤ 20 years of age) hospitalized for the primary and secondary admission diagnosis of COVID-19 and CHD from January 1, 2020 through December 31, 2020.

We used International Classification of Disease, Tenth Revision, Clinical Modification for diagnosis and procedure (ICD-10-CM, ICD-10-PR), and identified hospitalized children with COVID-19 infection, CHD and other variables studied. We excluded isolated anomalies of the peripheral vascular system from analysis. See supplementary Table 1 for ICD-10-CM, ICD-10-PR codes used. We categorized CHD patients into three groups according to their complexity: simple/mild, complex biventricular, and single ventricle CHD patients. Simple/mild CHD included atrial septal defect (ASD), ventricular septal defect (VSD), patent ductus arteriosus (PDA) and other septal defects. Single ventricle lesions included hypoplastic left heart syndrome, tricuspid atresia/stenosis, aortic atresia/stenosis and common ventricle. The remaining lesions were classified as biventricular complex lesions as per previous classification, which has been previously used in the pediatric cardiology literature [26]. The diagnosis, classification and ICD-10 codes of these patients are presented in supplemental Table 2.

The primary aim was to compare in-hospital mortality between children with COVID-19 infection with and without concomitant CHD. The secondary outcomes were serious cardiovascular and non-cardiovascular in-hospital complications. Diagnoses and procedures available for study in the NIS included myocarditis, tachyarrhythmias, heart block, sudden cardiac arrest, acute respiratory failure, acute respiratory failure requiring invasive mechanical ventilation (IMV) and non-invasive mechanical ventilation (NIMV), acute kidney injury, and need for ECMO. We examined the differences in the length of hospital stay between groups (CHD vs. non-CHD).

Descriptive and inferential statistics were performed using the NIS complex survey design, accounting for clusters, strata, and weighting. For continuous variables such as age and length of stay, median with interquartile range (IQR) were reported. Weight-adjusted Wilcoxon signed rank tests were used for non-evenly distributed continuous variables and Weight-adjusted Chi-square tests were used for categorical variables [21].

The statistical analysis methodology used in this study is reported in our previous publication [21]. In brief, the variables used in the multivariable analysis were selected after a rigorous review of the pediatric literature on COVID-19 infection and CHD. During selection, variables with reliable and consistent ICD codes were identified. For regression modeling, univariable analyses of each variable of interest were performed first, followed by a multivariable analysis incorporating additional variables (age, sex, race/ethnicity, asthma/reactive airway disease, prematurity, presence of respiratory and musculoskeletal congenital anomalies, the presence of chromosomal anomalies and zip code of patients’ home neighborhood)  to determine the effects of covariates and confounding variables on the outcome of interest. Zip code of patients’ home neighborhood is a quartile classification of the estimated median household income of residents in the patient’s ZIP Code. The quartiles are identified by lowest to highest, indicating the poorest to wealthiest populations. We performed logistic regression analysis for the odds ratios (ORs) of the risk of mortality and other complications mentioned above. Multiple linear regression analysis was used to assess differences in the length of hospital stay.

Weights provided by HCUP/NIS were used in all analyses to account for the complex sampling design and clustering for the analysis. All statistical analyses were performed using Stata statistical software (version 15.1), R version 3.6.0 [27] and R Studio 1.2. [28] Complex survey design of the NIS was accounted for using the *survey* package [29] and tables were generated using the *tableone* package [30], both in R.

## Results

We identified 36,690 pediatric COVID-19 admissions from January 1 to December 31, 2020, based on inclusion and exclusion criteria. Baseline characteristics of children with COVID-19 with and without CHD are presented in Table 1. Of the total population, 1240 (3.4%) children had CHD, with 155 (12.5%) having single ventricle pathology, 770 (62.1%) having mild lesions, and 315 (25.4%) diagnosed with biventricular complex lesions.

**Table 1:** Characteristics of pediatric patients with and without congenital heart disease (CHD) hospitalized with COVID-19 (N = 36,690)

Variables	CHD		
	Yes	No	P-value
Total hospitalized COVID-19 cases	1240	35,450	N/A
Age, years(median)	1 (IQR 0–5)	15 (IQR 3–19)	< 0.001
Length of stay(median)	5 (IQR 2–11)	3 (IQR 2–5)	< 0.001
Female	610(49.2%)	19,980 (56.4%)	0.025
Neighborhood Zip Codes according to family income			
Lowest 24th	495 (40.2%)	13,125 (37.4%)	P = 0.76
25-49th	295 (24.0%)	9170 (26.2%)	
50-74th	255 (20.7%)	7735 (22.1%)	
75th-100th	185 (15.0%)	5020 (14.3%)	
Race			
White	435 (37.8%)	10,490 (31.3%)	P = 0.20
Black	240 (20.9%)	7145 (21.3%)	
Hispanic	380 (33.0%)	12,620 (37.7%)	
Other	95 (8.3%)	3230 (9.6%)	
Prematurity < 37 weeks	80 (6.5%)	445 (1.3%)	< 0.001
Respiratory anomalies	60 (4.8%)	205 (0.6%)	< 0.001
Musculoskeletal anomalies	75 (6.0%)	380 (1.1%)	0.001
Chromosomal anomalies	260 (21.0%)	565 (1.6%)	< 0.001
Asthma/reactive airway disease	120 (9.7%)	5070 (14.3%)	0.043
Acute respiratory failure	410 (33.1%)	5820 (16.4%)	<0.001
Acute kidney injury	140 (11.3%)	1895 (5.3%)	< 0.001
Invasive mechanical ventilation(IMV)	205 (16.5%)	1795 (5.1%)	< 0.001
Non-invasive mechanical ventilation(NIMV)	120 (9.7%)	1055 (3.0%)	< 0.001
Tachyarrhythmias	60 (4.8%)	565 (1.6%)	< 0.001
Heart block	60 (4.8%)	340 (1.0%)	< 0.001
Sudden cardiac arrest	15 (1.2%)	155 (0.4%)	0.07
Myocarditis	<11^+^	510 (1.4%)	NS
ECMO^#^	15 (1.2%)	100 (0.3%)	0.05
In-hospital mortality	15 (1.2%)	295 (0.8%)	0.50

* Comparisons are done by Chi-square test. NS = not significant

^#^Extracorporeal membrane oxygenation

^+^Numbers < 11 are not reportable as per Healthcare Cost and Utilization Project (HCUP) guidelines; statistical testing is reported as significant or non-significant (NS, P > 0.05)

The overall in-hospital mortality rate for children hospitalized with COVID-19 infection was 0.8% (n = 310). Acute respiratory failure was present in 17.0% (n = 6230), acute kidney injury in 5.5% (n = 2035) and invasive mechanical ventilation in 5.5% (n = 2000).

Children with CHD were much younger than those who did not have underlying CHD [1 year (IQR 0–5) vs. 15 (IQR 3–19) years, p < 0.001], and stayed in the hospital longer than those without CHD [5 days (IQR 2–11) vs. 3 (IQR 2–5), p < 0.001].

In-hospital mortality was 1.2% (n = 15) in children with COVID-19 infection and CHD, this was 0.8%(n = 295) for those without CHD, P = 0.63. As shown in Table 2, after adjusting for age, sex, race, neighborhood zip codes, the presence of chronic medical conditions specifically asthma/reactive airway disease, and chromosomal anomalies, a multivariable regression model found that the risk of mortality in patients with CHD was not statistically significant, adjusted OR (aOR) of 1.7 (95% CI: 0.6–5.3), P = 0.32. In this model, older children (aOR: 1.1, 95% CI:1.1–1.1) had a higher risk of mortality while female sex had a lower risk (aOR: 0.4, 95% CI: 0.2–0.7).

**Table 2:** A multivariable logistic regression model to assess the risks of complications among children with congenital heart disease (CHD) hospitalized with COVID-19 infection in comparison with those children with COVID-19 and without CHD

Complications	Unadjusted Odds ratio with 95% CI	P-value	Adjusted Odds ratio with 95% confidence interval	P-value
Acute respiratory failure	2.5 (1.9–3.3)	< 0.001	2.0 (1.5–2.8)	< 0.001
Acute kidney injury	2.3 (1.5–3.4)	< 0.001	3.4 (2.2–5.4)	< 0.001
Invasive mechanical ventilation(IMV)	3.7 (2.6–5.3)	< 0.001	2.6 (1.6-4.0)	< 0.001
Non-invasive mechanical ventilation (NIMV)	3.5 (2.2–5.4)	< 0.001	2.7 (1.4–5.2)	0.002
Tachyarrhythmias	3.1 (1.7–5.8)	< 0.001	4.2 (1.8–9.9)	< 0.001
Heart block	5.3 (2.8–9.8)	< 0.001	5.0 (2.4–10.8)	< 0.001
Sudden cardiac arrest	2.8 (0.8–9.2)	0.092	1.7 (0.6–4.7)	0.32
Myocarditis	0.6 (0.1–2.3)	0.41	0.4 (0.1–3.3)	0.41
ECMO	4.3 (1.3–14.6)	0.018	3.9 (0.9–17.4)	0.07
In-hospital mortality	1.5 (0.5–4.7)	0.63	1.7 (0.6–5.3)	0.32

Data presented as adjusted odds ratio (95% confidence interval) after being adjusted for age, sex, race/ethnicity, neighborhood zipcodes as well as asthma/reactive airway disease, prematurity, chromosomal, musculoskeletal, and respiratory tract anomalies

Similarly, we performed univariable and multivariable regression analyses to compare the risk of cardiovascular complications, including tachyarrhythmias, heart block, myocarditis and sudden cardiac arrest and need for ECMO. Children admitted with COVID-19 with underlying CHD had higher risk of tachyarrhythmias (aOR = 4.2, 95% CI: 1.8–9.9 and bradyarrhythmias (aOR = 5.0, 95% CI: 2.4–10.8, while they were not at increased risk of myocarditis, need for ECMO and sudden cardiac arrest (Table 2). Regarding serious but non-cardiovascular complications, children with CHD had increased risk of acute respiratory failure aOR = 2.0 (1.5–2.8), P < 0.001, acute respiratory failure requiring invasive mechanical ventilation aOR = 2.6(1.6-4.0), P < 0.001 and non-invasive ventilation aOR = 2.7 (1.4–5.2) P = 0.002, and acute kidney injury aOR = 3.4 (2.2–5.4), P < 0.001, compared to those without CHD (Table 2).

When comparing outcomes according to the CHD category (mild/simple, biventricular complex and single ventricle lesions), there were no statistical differences in in-hospital mortality, cardiovascular and non-cardiovascular complications (Supplemental Tables 2 and 3). Inability of the statistical tests to reach significant differences could be potentially from small sample size(power). Given the relatively smaller sample size of the subgroup of CHD patients and the outcome variables, and the HCUP guidelines for protecting individual patient identities which dictate that counts of < 11 cannot be reported, individual data are censored from this report.

## Discussion

This is one of the largest studies on hospitalized children with CHD and COVID-19 infection. The NIS 2020 dataset is a large national database of more than 36,000 pediatric COVID-19-associated hospitalizations, including 1240 children with CHD. We report the following major findings: (1) children with CHD were more likely to have severe cardiovascular and non-cardiovascular complications like tachyarrhythmia, conduction abnormalities, acute respiratory failure, respiratory failure requiring invasive and non-invasive ventilation and acute kidney injury; (2) length of hospital stay was longer in children with CHD compared to those without CHD. In agreement with our findings, Ehwerhemuepha et al. have used Cerner EMR Data (a large, multicenter electronic health records database, which includes both inpatient and outpatient data) and found that children with CHD had higher risk of severe COVID-19 infection, which was defined as needing oxygen supplementation or dying during hospitalization [2]. However they did not directly compare the outcomes amongst hospitalized children with COVID-19 with and without CHD, thus our study adds specific information regarding this subgroup of children with more severe COVID-19 with the advantage of including an estimated 98% of all inpatient admissions.

In our study, in-hospital mortality rates in COVID-19 related hospitalizations with CHD is 50% higher compared to those without CHD (1.2% vs. 0.8%). Although the adjusted risk of mortality in CHD was 1.7 times higher, the association was not statistically significant likely due to lack of power(95% CI 0.6–5.3), P = 0.32. Reports with a smaller number of children with CHD have reported a higher percentage of death in children with CHD and COVID-19 infection [31, 32]. In a study of 160 children with CHD hospitalized with COVID-19, Strah et al. reported a mortality rate in children with COVID-19 of 3.8% vs. 0.8% in the non-CHD group. The length of stay was longer with mean and standard deviation of 22.2土42.7 days, compared to 12.3土24 days in ours, suggestive of higher illness severity in their cohort [31]. In the study by Downing et al. [33], there were 54 patients in the 1–17 year age group and there was no mortality in this group. Both complex and non-complex CHD groups had higher mortality rates compared to non-CHD cohorts and also the higher mortality was noted among adults. Interestingly, non-complex CHD had higher mortality risk than severe CHD.

In a previous report on influenza-related hospitalizations using the KID database from 2003 to 2016, we reported 0.5% in-hospital mortality from influenza-related illnesses in a similar cohort [21]. In 2020, mortality was higher in COVID-related hospitalization when compared to those in influenza in previous years. Important explanation is the differences in these two RNA viruses and their surface antigen compositions which may be partially responsible for the observation that COVID-19 seems to produce more severe disease compared to influenza. This could be partially attributed to the lack of COVID-19 vaccination for children during our study period, while vaccination for influenza has been available and has been shown to decrease hospitalization and mortality [34].

In this study, children with CHD had higher rates of chromosomal anomalies than those without CHD, 21.0% (n = 260) vs. 1.6% (n = 565). Children with chromosomal anomalies often have comorbid conditions not reflected in this dataset that might increase their risk of complications from COVID-19. However, morbidity differences remained after adjusting for chromosomal anomalies on logistic regression models for primary and secondary outcomes.

The risk of cardiovascular complications, including tachyarrhythmias and heart block were significantly higher in CHD children than those without CHD in our study. COVID-19 infection has previously been reported to affect the conduction pathway causing mostly first-degree heart block though some progress into higher degrees of heart block [35]. The etiology of the conduction abnormalities in COVID-19 infection remains unclear but may result from inflammation and edema of the conduction tissue as part of a more diffuse process of myocardial injury [36–39]. It is possible that in patients with CHD, and particularly repaired CHD, that there is a predisposition to arrhythmias or an increase in susceptibility to inflammation during COVID-19 infection due to underlying conduction and myocardial abnormality from prior injury or scarring.

In children with COVID-19, the risk of myocarditis was similar between those with and without CHD. Although the exact mechanism of how coronavirus could affect the heart is not precisely known, a recent study has revealed that certain viral proteins in SARS-CoV-2 seem to cause direct cardiac tissue injury [40]. Another plausible cause has been hypoxia, which, if prolonged due to the acute respiratory illness could lead to anaerobic metabolism, metabolic acidosis and free oxygen radical production, which subsequently could lead to myocardial injury [41].

The data presented here was from the initial phase of COVID-19 pandemic when the vaccine against COVID-19 infection was not yet available or approved in children. Since 2020, millions of children 6 months and older have received the vaccine. The studies have reported on vaccine efficacy in preventing the complications and mortality related to covid-19 hospitalization [16–19] but it remains to be seen whether this vaccine effect will extend to children with CHD in decreasing mortality and length of stay.

We used the NIS database, which provided a larger sample size based on population sampling of hospitalized pediatric patients with COVID-19, which was the main strength of this study. However, there were multiple limitations to this approach. First, this database includes only hospitalized children, who are most likely to have severe disease from COVID-19 infection. So, the findings may not be applicable to the outpatient settings and to those children with chronic cardiac disease who were not hospitalized. Second, incorrect or missing information may potentially exist in medical coding and billing databases, such as NIS. Third, there was no information on the accuracy of the diagnosis, although NIS uses very stringent protocols and methodologies to provide accurate data before the database is released. Also, parsing COVID-19 admissions (either with or for COVID-19) from ICD-10 codes in databases like NIS is challenging. Lastly, we were not able to study the influence of vaccination status on clinical outcomes using this database as the vaccines for children were not available or approved during the study period.

## Conclusions

In conclusion, our findings indicate that children with CHD who were admitted with COVID-19 infection prior to widespread availability of vaccines were at increased risk of severe cardiovascular and non-cardiovascular adverse clinical outcomes. Children with CHD admitted with COVID-19 had increased length of hospital stay and utilization of healthcare resources versus those without CHD. Future studies to examine the effect of COVID-19 vaccination on the outcomes of children with chronic conditions like CHD are needed.

## Electronic supplementary material

Below is the link to the electronic supplementary material.

Supplementary Material 1

## Data Availability

Because of limitations of the NIS data use agreement and availability of the data directly from the Agency for Healthcare Research and Quality, the data, analytic methods, and study materials will not be made available to other researchers for purposes of reproducing the results or replicating the procedure. Please contact hcup@ahrq.gov to obtain a copy of the database and the process to obtain the database.

## Abbreviations

CHD: Congenital heart disease
COVID-19: Coronavirus Disease due to SARS-CoV-2 virus
AHRQ: Agency for Healthcare Research and Quality
HCUP: Healthcare Cost and Utilization Project
NIS: National Inpatient Sample
