# Supplementary material for: Impact of congenital heart disease on outcomes among pediatric patients hospitalized for COVID-19 infection
Source: BMC Pediatr. 2023 May 16;23:240. doi: 10.1186/s12887-023-04058-2 (PMC10185948; doi:10.1186/s12887-023-04058-2)
Supplement: Supplementary file 1 — Supplementary Material 1 [file 12887_2023_4058_MOESM1_ESM.docx]

**Supplementary Table 1.** Variables and the corresponding ICD-10 codes.

| **Variables** | **ICD-10 code** |
| --- | --- |
| ***Procedures codes*** | |
| **Invasive mechanical ventilation(IMV)** | 0DH57BZ,0DH58BZ, 0BH17EZ, 0BH18EZ, 0B717DZ, 0B718DZ, 0BH07DZ, 0WHQ7YZ, 5A1935Z, 5A1945Z, 5A1955Z |
| **Non-invasive mechanical ventilation(NIMV)** | 5A09357, 5A09457, 5A09557, 5A09358, 5A09458, 5A09558 |
| **Extracorporeal membrane oxygenation** | 5A1522F, 5A1522G, 5A1522H |
| ***Diagnostic codes*** | |
| **COVID-19** | U07.1, B97.29 |
| **Congenital heart disease** | Q20.xx to Q26.xx |
| **Myocarditis** | I51.4, I40.8, I40.9, B33.20, I40.1, I40.0, J10.82, I41, B3322, I40 |
| **Acute respiratory failure** | J96.xx, J80, R09.2, J95.82x |
| **Acute kidney injury** | N17, N17.0, N17.1, N17.2, N17.8, N17.9 |
| **Tachyarrhythmias** | I47.1, I47.2, I49.01, I49.0, I48.0, I48.3, I48.4 |
| **Heart block** | I44.1, I44.2 |
| **Sudden cardiac arrest** | I46.x |
| **Asthma/reactive airway disease** | J45.xx |
| **Prematurity (<37 weeks of gestation)** | P072.1 to P072.6, P073.x |
| **Congenital musculoskeletal anomalies** | Q65.xx-Q79.xx |
| **Congenital respiratory tract anomalies** | Q30.xx-Q34.xx |
| **Chromosomal anomalies** | Q90.xx-Q99.xx |

**Supplementary Table 2:** Categories of congenital heart disease (CHD).

1=Single ventricle

2=Other complex CHD

3=Simple CHD(atrial or ventricular septal defect, patent ductus arteriosus)

| Category | CHD lesion | ICD-10 |
| --- | --- | --- |
| 1 | Common ventricle | Q20.4 |
| 1 | Tricuspid atresia and stenosis | Q22.4, Q22.9 |
| 1 | Hypoplastic left heart syndrome | Q23.4 |
| 1 | Atresia and stenosis of aorta | Q25.2, Q25.3 |
| 2 | Common truncus | Q20.0 |
| 2 | Complete transposition of great vessels | Q20.3 |
| 2 | Double outlet right ventricle | Q20.1 |
| 2 | L-transposition of the great arteries | Q20.5 |
| 2 | Tetralogy of Fallot | Q21.3 |
| 2 | Cor biloculare | Q20.8 |
| 2 | Pulmonary valve anomaly, unspecified | Q22.3 |
| 2 | Pulmonary atresia | Q22.0 |
| 2 | Congenital pulmonary stenosis | Q22.1 |
| 2 | Other anomalies of pulmonary valve | Q22.2 |
| 2 | Ebstein's anomaly | Q22.5 |
| 2 | Congenital stenosis of aortic valve | Q23.0 |
| 2 | Congenital insufficiency of aortic valve | Q23.1 |
| 2 | Congenital mitral stenosis | Q23.2 |
| 2 | Congenital mitral insufficiency | Q23.3 |
| 2 | Subaortic stenosis | Q24.4 |
| 2 | Cor triatriatum | Q24.2 |
| 2 | Infundibular pulmonic stenosis congenital | Q24.3 |
| 2 | Obstructive anomalies of heart not elsewhere classified | Q24.8 |
| 2 | Coronary artery anomaly congenital | Q24.5 |
| 2 | Malposition of heart and cardiac apex | Q24.0 |
| 2 | Other congenital anomalies of heart | Q24.8 |
| 2 | Unspecified anomaly of heart | Q20.9, Q24.9 |
| 2 | Coarctation of the aorta | Q25.1 |
| 2 | Interruption of aortic arch | Q25.2 |
| 2 | Other congenital anomalies of aorta | Q25.4 |
| 2 | Anomalies of aortic arch | Q25.4 |
| 2 | Other anomalies of aorta | Q25.4 |
| 2 | Congenital anomalies of great veins, unspecified | Q26.9 |
| 2 | Total anomalous pulmonary venous return | Q26.2 |
| 2 | Partial anomalous pulmonary venous connection | Q26.3 |
| 2 | Other anomalies of great veins  (Scimitar or left superior vena cava) | Q26.8 |
| 3 | Ventricular septal defect | Q21.0 |
| 3 | Ostium secundum atrial septal defect | Q21.1 |
| 3 | Endocardial cushion defect, unspecified type | Q21.2 |
| 3 | Ostium primum atrial septal defect | Q21.2 |
| 3 | Other bulbus cordis anomaly of septal defect | Q20.8, Q21.8 |
| 3 | Unspecified defect of septal closure | Q21.9 |
| 3 | Patent ductus arteriosus | Q25.0 |

**Supplementary table 3**. Characteristics of pediatric patients in different categories of congenital heart disease (CHD) hospitalized with COVID-19.

| Variables | Categories of CHD | | |  |
| --- | --- | --- | --- | --- |
|  | Mild CHD | Biventricular complex lesions | Univentricular lesions | P-value |
| Total hospitalized covid-19 cases | 770 | 315 | 155 | NA |
| Age, years(median) | 0 (IQR 0-4) | 1 (IQR 0-7.5) | 2.0 (IQR 0-10.8) | 0.047 |
| Length of stay(median, IQR) | 5 (IQR 2-10) | 5.5 (IQR 2-16.8) | 5.5 (IQR 2.0-12.5) | 0.77 |
| Female | 405 (52.6%) | 115 (36.5%) | 90 (58.1%) | 0.077 |
| Prematurity <37 weeks | 75 (9.7%) | ✝️ | ✝️ | NS |
| Respiratory anomalies | 40 (5.2%) | ✝️ | ✝️ | NS |
| Musculoskeletal anomalies | 35 (4.5%) | 40 (12.7%) | ✝️ | <0.05 |
| Chromosomal anomalies | 175 (22.7%) | 85 (27.0%) | ✝️ | NS |
| Asthma/reactive airway disease | 75 (9.7%) | 35 (11.1%) | ✝️ | NS |
| Acute respiratory failure | 255 (33.1%) | 100 (31.7%) | 55 (35.5%) | 0.93 |
| Acute kidney injury | 70 (9.1%) | 45 (14.3%) | 25 (16.1%) | 0.36 |
| Invasive mechanical ventilation(IMV) | 110 (14.3%) | 65 (20.6%) | 30 (19.4%) | 0.55 |
| Non-invasive mechanical ventilation(NIMV) | 75 (9.7%) | 25 (7.9%) | 20 (12.9%) | 0.72 |
| Tachyarrhythmia | 25 (3.2%) | ✝️ | 25 (16.1%) | <0.05 |
| Heart block | 20 (2.5%) | 30 (9.5%) | ✝️ | NS |
| Sudden cardiac arrest | ✝️ | ✝️ | ✝️ | NS |
| Myocarditis | ✝️ | ✝️ | ✝️ | NS |
| ECMO^#^ | ✝️ | ✝️ | ✝️ | NS |
| In-hospital mortality | ✝️ | ✝️ | ✝️ | NS |

* Comparisons are done by Chi-square test)

^#^Extracorporeal membrane oxygenation

✝️ Numbers <11 are not reportable as per Healthcare Cost and Utilization Project (HCUP) guidelines; statistical significance testing is reported as significant or non-significant (NS, >0.05).
